# Inhibition of human endogenous retrovirus-K by antiretroviral drugs

**DOI:** 10.1186/s12977-017-0347-4

**Published:** 2017-03-22

**Authors:** Richa Tyagi, Wenxue Li, Danelvis Parades, Mario A. Bianchet, Avindra Nath

**Affiliations:** 1 Section of Infections of the Nervous System, National Institute of Neurological Disorders and Stroke, National Institutes of Health, Room 7C-103, Bldg 10, 10 Center Drive, Bethesda, MD 20892 USA; 2 Department of Neurology, Johns Hopkins University, Baltimore, MD USA; 3 Department of Biophysics and Biophysical Chemistry, Johns Hopkins University, Baltimore, MD USA

**Keywords:** Human endogenous retrovirus-K, HIV, Antiretroviral, Amyotrophic lateral sclerosis, Comparative modeling

## Abstract

**Background:**

Human endogenous retroviruses (HERVs) are genomic sequences of retroviral origin which were believed to be integrated into germline chromosomes millions of years ago and account for nearly 8% of the human genome. Although mostly defective and inactive, some of the HERVs may be activated under certain physiological and pathological conditions. While no drugs are designed specifically targeting HERVs, there are a panel of antiretroviral drugs designed against the human immunodeficiency virus and approved by the Federal Drug Administration (FDA).

**Results:**

We determined if these antiretroviral drugs may also be effective in inhibiting HERVs. We constructed a plasmid with consensus HERV-K sequence for testing the effect of antiretroviral drugs on HERV-K. We first determined the effects of nucleoside and non-nucleotide reverse transcriptase (RT) inhibitors on HERV-K by product enhanced reverse transcription assay. We found that all RT inhibitors could significantly inhibit HERV-K RT activity. To determine the effects of antiretroviral drugs on HERV-K infection and viral production, we pseudotyped HERV-K with VSV-G and used the pseudotyped HERV-K virus to infect HeLa cells. HERV-K production was measured by quantitative real time polymerase chain reaction. We found that RT inhibitors Abacavir and Zidovudine, and integrase inhibitor Raltegravir could effectively block HERV-K infection and production. However, protease inhibitors were not as effective as RT and integrase inhibitors.

**Conclusions:**

In summary, we identified several FDA approved antiretroviral drugs that can effectively inhibit HERV-K. These antiretrovirals may open new prospects for studying HERV-K pathophysiology and potentially for exploring treatment of diseases in which HERV-K has been implicated.

## Background

Tremendous progress has been made in the development of antiretroviral drugs that target HIV replication. Initial drugs targeted reverse transcriptase (RT), however now drugs are available that target all major parts of the viral life cycle. This includes blockage at viral entry, integrase (IN) inhibitors that prevent integration of proviral DNA into the chromosomal DNA, protease inhibitors that prevent cleavage of the Gag–Pol polyprotein and maturation inhibitors. However, the effect of these drugs on endogenous retroviruses (ERV) remains unknown. ERV’s constitute nearly 8% of the human genome. While they play an important role in embryonic development [1], they remain silent in adults. Under pathological circumstances, these viral elements may get reactivated. For example, we previously showed that in patients with amyotrophic lateral sclerosis (ALS), human ERV-K (HERV-K) was expressed and this activation causes neurotoxicity [2, 3]. Hence it would be important to know if inhibition of HERV-K could alter the course of ALS. HERV-K activation has also been associated with schizophrenia and some cancers [4–7]. HERV-K is a beta-retrovirus. It has similarity to lentivirus HIV including 5′ and 3′ LTR region, an envelope (*env*), *gag* and *pol* genes. HERV-K is the most recently acquired ERV in the human genome and hence has several intact open reading frames [8]. The *pol* gene encodes RT, protease and IN. Rec protein is encoded from an alternative spliced messenger RNA from *env* and is similar in function to Rev protein of HIV. In some HERV-K sequences there is a deletion of 292 base pairs at the *pol*–*env* junction as a result Rec is not formed [8]. This resulted in an mRNA for a ~9 kDa fusion protein referred to as Np9, the function of which is not yet understood. HERV-K lacks the other regulatory genes such as *tat*, *nef*, *vpr* and *vpu* which are present in the HIV genome. Since HERV-K has its own RT, protease and IN, we screened a panel of FDA approved anti-HIV drugs that target these enzymes, for their ability to inhibit HERV-K.

## Results

### Viral particle production with consensus HERV-K genome

**Fig. 1:**
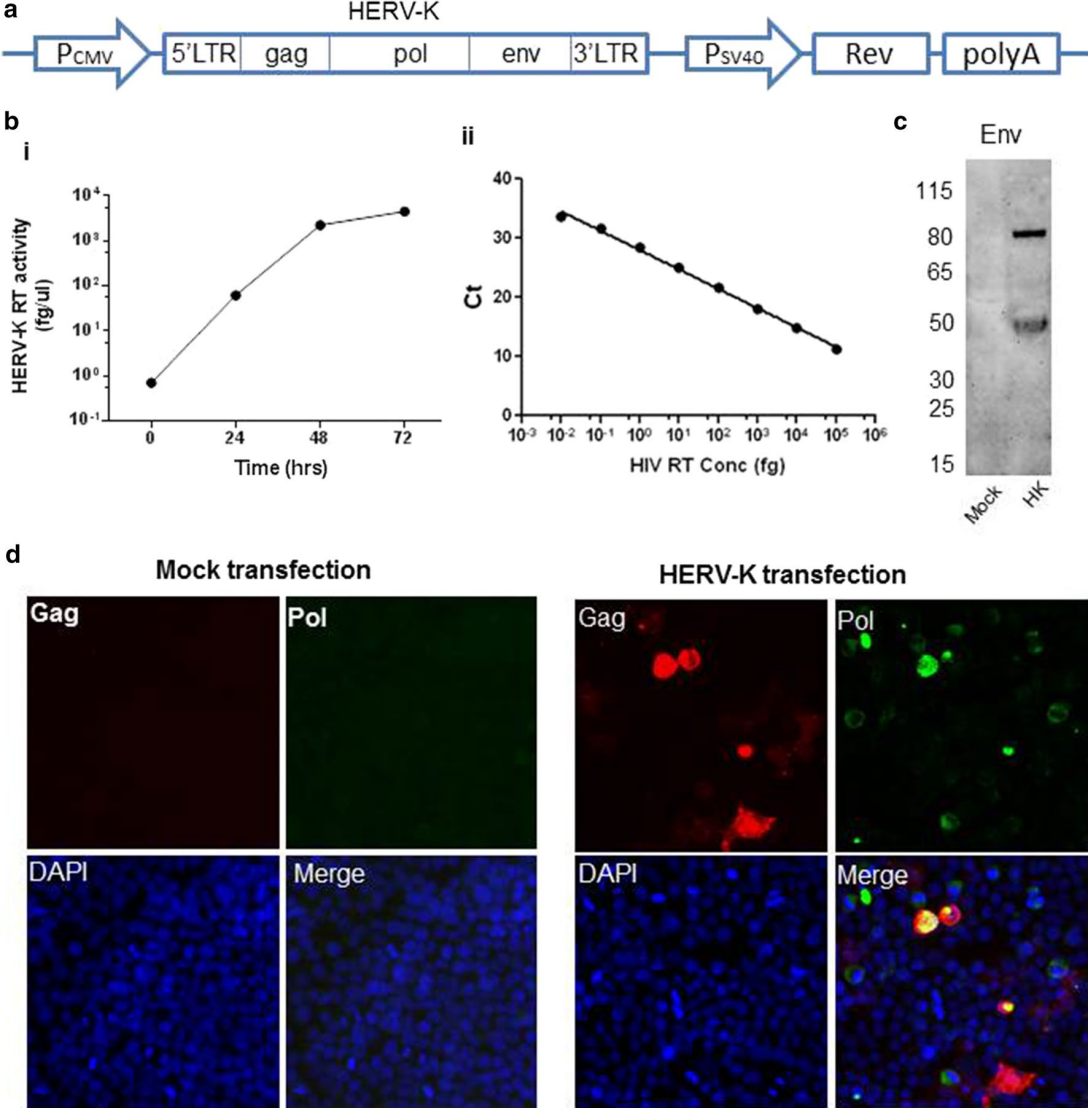
Consensus HERV-K has the ability to generate active viral particles. **a** The consensus complete HERV-K genomic sequence was cloned into the pcDNA3.1 vector with HIV-1 Rev resulting in a plasmid termed pCD-HK/Rev. **bi** HeLa cells were transfected with the pCD-HK/Rev plasmid in combination with plasmids for HIV-1 Tat. The reverse transcriptase (RT) activity in the culture supernatant was determined by PERT assay at 24, 48 and 72 h post-transfection. **ii** Recombinant HIV RT was diluted serially in culture media and used as an activity standard. HERV-K RT activity in **i** was quantified using this standard. **c** Western blot analyses for HERV-K Env expression in 293T cells after transfection with pCD-HK/Rev. **d** HERV-K transfected or mock transfected cells were fixed 24 h post-transfection and immunostained with primary antibodies for HERV-K Gag and HERV-K Pol. Alexa 488-conjugated goat anti-rabbit IgG and Alexa 594-conjugated goat anti-mouse IgG were used as secondary antibodies

To generate HERV-K viral particles for determining the effects of antiretroviral drugs on HERV-K, a consensus HERV-K sequence [9] was synthesized and subcloned into pcDNA 3.1 vector. Because HIV-1 Rev can significantly enhance the transcription of HERV-K viral gene, an HIV-1 Rev expression cassette was also inserted into the construct which we have termed pCD-HK/Rev (Fig. 1a). After transfection of either HeLa or 293T cells with pCD-HK/Rev and pCD-Tat plasmid, the production of viral particles was confirmed by electron microscopy [9]. The amount of viral production was quantified by measuring RT activity in the culture supernatant with PERT assay (Fig. 1bi). Recombinant HIV-1 RT was used as a positive control and to make a standard curve (Fig. 1bii). The HERV-K RT quantification using this standard curve is only relative to HIV RT, not an absolute RT activity. HERV-K viral particles released to the culture media increased until 48 h after transfection, and plateaued at 72 h. To further confirm HERV-K viral gene expression, cell lysate was collected 48 h post-transfection. The expression of HERV-K Env and Gag was determined by Western blot analysis (Figs. 1c, 7d). The Gag antibody recognized both precursor Gag (70 kDa) and mature Gag (27 kDa) proteins [10]. The Env antibody recognized both full-length (80 kDa) Env and the transmembrane subunit (42 kDa). We further immunostained HERV-K Gag and Pol in HERV-K plasmid transfected 293T cells (Fig. 1d). As shown in the “HERV-K transfection” panel, some cells co-expressed HERV-K Gag and Pol while other cells expressed only Gag or Pol.

### Direct inhibition of HERV-K reverse transcriptase (RT) by HIV-1 RT inhibitors

**Fig. 2:**
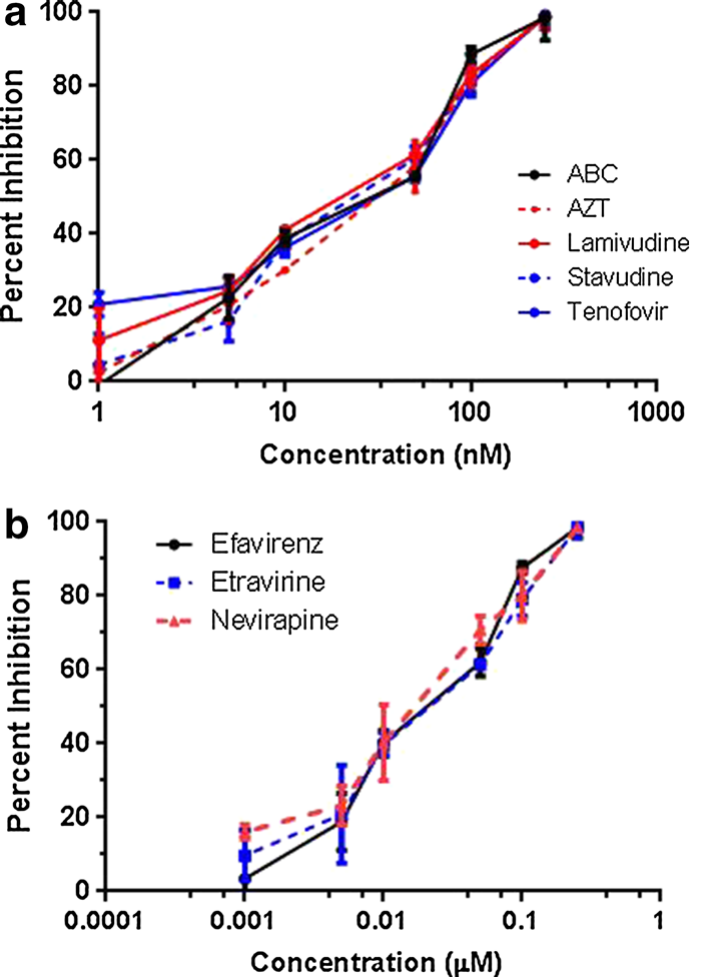
HIV reverse transcriptase inhibitors can inhibit HERV-K reverse transcriptase. HERV-K supernatant was collected from Hela cells transfected with pCD-HK/Rev plasmid in combination with HIV-1 Tat. HIV-RT inhibitors: **a** nucleoside RT inhibitors or **b** non-nucleotide RT inhibitors were added in a dose ranging from 0.05 to 0.25 µM to collected supernatant and PERT assay was performed to quantify HERV-K RT. Any change compared to no treatment was reported as percent inhibition

We first tested the effects of HIV-1 RT inhibitors on HERV-K RT enzyme activity in a cell-free system. Viral particles were harvested from either HeLa or 293T culture media after transfection with HERV-K plasmid. HERV-K RT was then released from culture media by treatment with Triton X-100. PERT assay was used to determine the activity of HERV-K RT. Serial dilutions of inhibitors were added to the extracted HERV-K RT just prior to the PERT assay. We tested the following nucleotide RT inhibitors: tenofovir, abacavir, stavudine, lamivudine, and zidovudine. As shown in Fig. 2a, all nucleotide RT inhibitors showed significant and dose-dependent inhibition of HERV-K RT. They had similar dosage-response curves and IC_90_ values. We also tested non-nucleotide inhibitors efavirenz, etravirine, and nevirapine on HERV-K RT. These drugs also showed significant inhibition of HERV-K RT activity with similar IC_90_ values (Fig. 2b).

### Inhibition of HERV-K by HIV-1 RT inhibitors

**Fig. 3:**
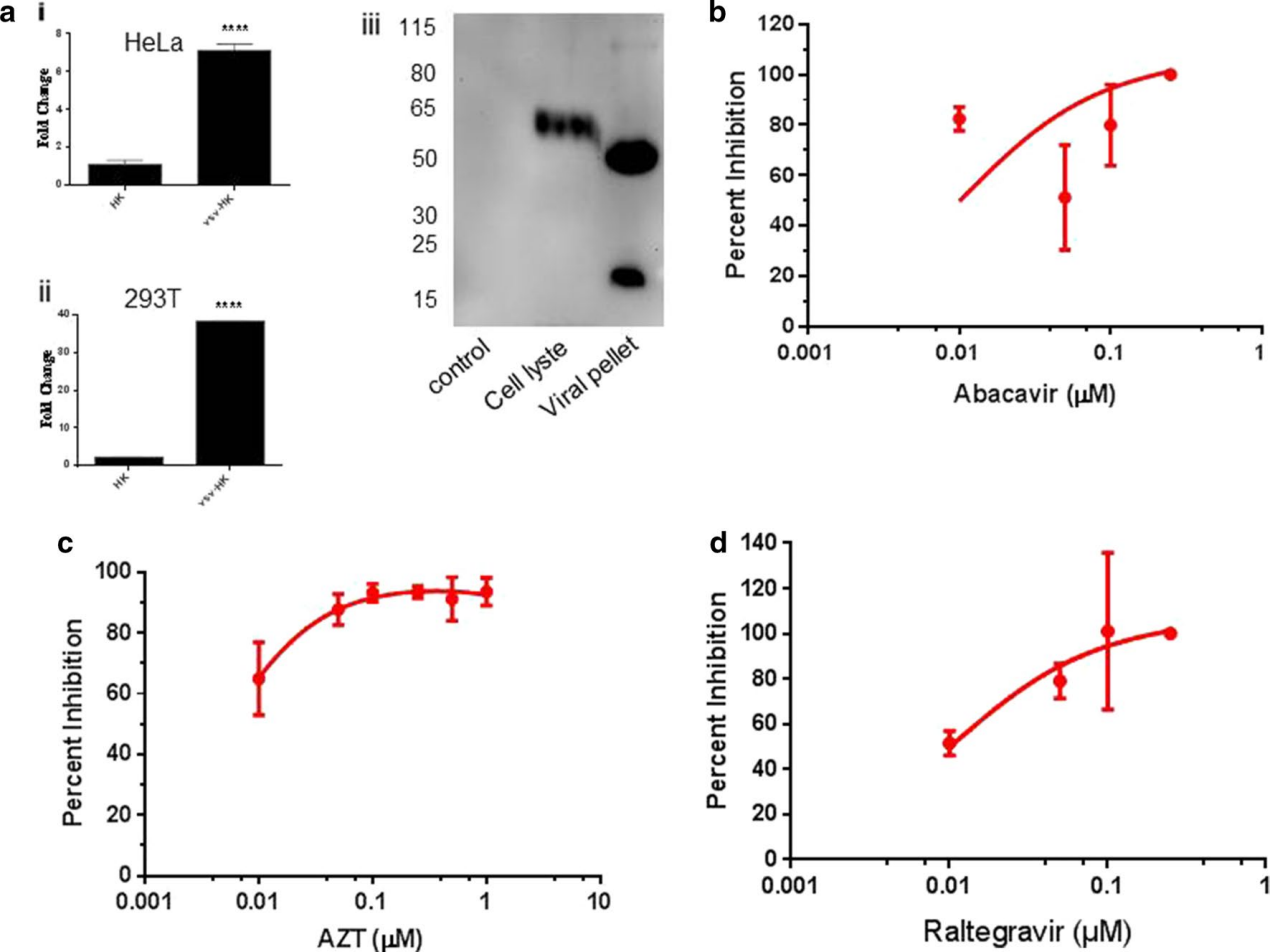
HERV-K can be effectively inhibited by Abacavir, AZT and Raltegravir: **ai** Hela cells and **ii** 293T cells were infected with HERV-K (HK) or VSV-G pseudotyped HERV-K (vsv-HK) viral particles. Total RNA was extracted 6 days post-infection and quantitative PCR was used to determine HERV-K gag mRNA expression. Glyceraldehyde 3-phosphotate dehydrogenase (GAPDH) was used as internal control and titers were expressed as fold change. **iii** Western blot of VSV-G protein to show that VSV-G protein is incorporated into the HERV-K viral particles. **b**–**d** Hela cells were infected with 80 pg of VSV-G pseudotyped HERV-K virus and treated with **b** Abacavir, **c** Zidovudine (AZT) or **d** Raltegravir, in a dose ranging from 0.05 to 0.25 µM. Six days post infection gag mRNA expression was quantified using quantitative PCR. Gag expression was compared to no treatment as control and expressed as percent inhibition. Data represent mean ± SEM of at least 3

To determine the effects of RT inhibitors on HERV-K infection, we generated HERV-K viral particles with VSV-G pseudotype to facilitate infection of HERV-K in HeLa cells. VSV-G pseudotyped HERV-K viral particles allow efficient infection of most cell types while replication of HERV-K inside the cells is not altered by VSV-G protein. To normalize the amount of viral particles used for infection, recombinant HIV-1 RT was used as equivalent of HERV-K RT to generate a standard curve for the PERT assay. HERV-K viral particles were then expressed as the amount of equivalent RT. Infection of HERV-K and viral production was determined by quantitative polymerase chain reaction (qPCR) for the *gag* gene. As shown in Fig. 3ai, after 6-days of infection in HeLa cells, the level of *gag* expression with VSV-G pseudotyped HERV-K showed a sevenfold increase compared to that of without pseudotyping. Similarly, in the 293T cells, the level of gag expression with VSV-G pseudotyping showed a 36-fold increase compared to that of without pseudotyping (Fig. 3aii). Western blot analysis further confirmed that VSV-G protein is incorporated into the HERV-K viral particles (Fig. 3aiii), however, VSV-G protein was further processed when it was incorporated into the viral particle, as indicated by a different molecular mass of the protein compared to that in the cell lysate. To test the effects of RT inhibitors on HERV-K infection, RT inhibitors were added to the cell culture medium immediately after the inoculation of VSV-G pseudotyped HERV-K virus. The concentration of inhibitors was chosen such that they did not cause toxicity to HeLa cells as determined by a cell viability assay. After 6 days of infection, HERV-K *gag* RNA was determined by qPCR as a measurement of HERV-K production. Abacavir (Fig. 3b) and Zidovudine (Fig. 3c) both inhibited HERV-K in a dose-dependent manner, with IC_90_ of 0.175 and 0.070 µM respectively.

### Comparative modeling of HERV-K RT

**Fig. 4:**
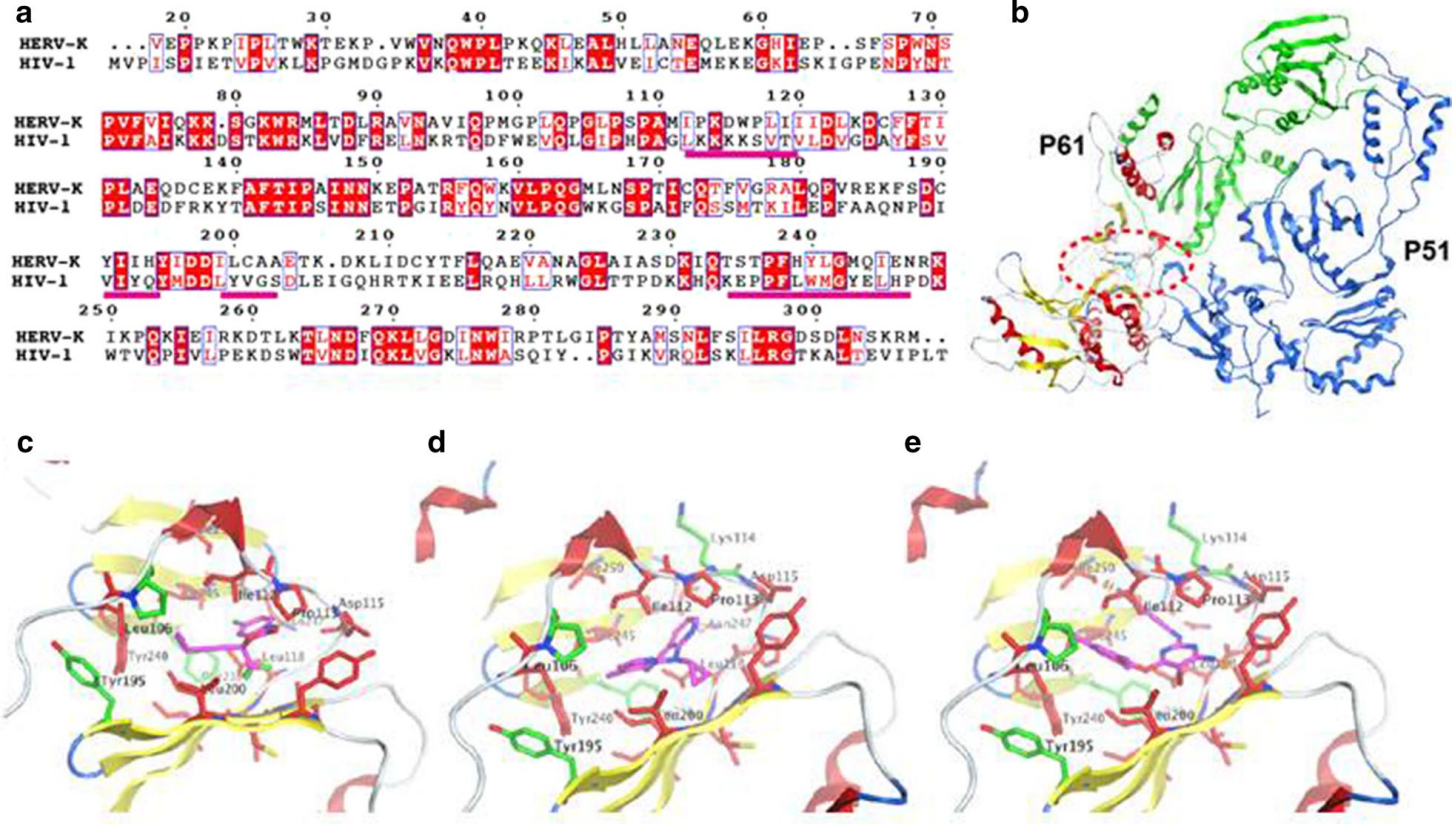
Comparative modeling of HERV-K reverse transcriptase (RT) based on HIV RT. **a** Sequence alignment between the target and the template (4WE1). **b** Final Model of HERV-K RT showing P51 and P66 subunits. The *red oval* indicates the NNRTI-binding region. **c**–**e** Efavirenz, Nevirapine, and Etravirine, respectively bound to the NNRTI-binding pocket. Non conserved residues are colored *red*, *green* the conserved between HERV-K and HIV-1 RT. The NNRTIs are *colored* with their carbon atom in *magenta*. The *magenta underlined* sequence are residues lining the NNRTI-binding pocket

HERV-K RT displays 21.5% sequence identity with HIV-1 RT (Fig. 4a). Comparative modeling of HERV-K RT (Fig. 4b) and its complexes with NNRTIs is shown using HIV-1 RT complex with Efavirenz (1JKV, Fig. 4c) and Nevirapine (E3QIP; Fig. 4d) as templates. The complex with etravirine (Fig. 4e) was modeled ab initio using the others as guides. These drugs bind to an allosteric site in a hydrophobic cavity (NNRTI binding pocket) nearby the RT motif YIDD that interacts with the DNA. The model shows that most of the residues that line the cavity are not conserved. However, the small inhibitors Efavirenz and nevirapine (Fig. 4c, d, respectively) still can be docked snuggly inside the cavity. The large etravirine showed steric clashes of its benzonitrile side chain and central ring amine substituent with the RT. However, with small protein adjustment, etravirine could still fit within the cavity.

### Inhibition of HERV-K by HIV-1 protease inhibitors

**Fig. 5:**
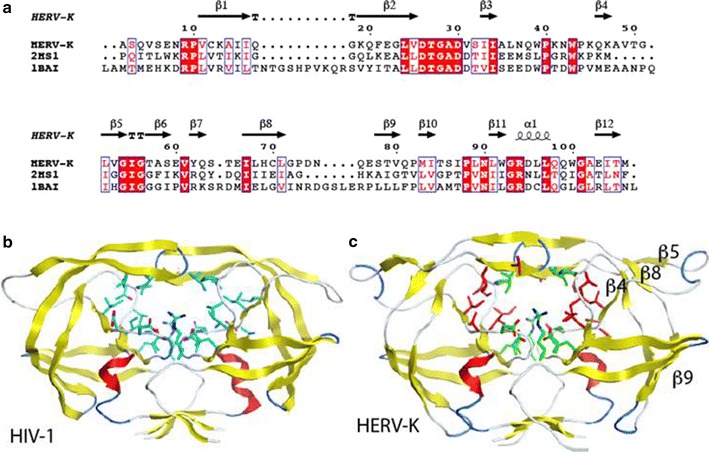
Modeling of HERV-K protease: **a** alignment of HERV-K with HIV-1 protease (2HS1) and Rous sarcoma virus protease (1BAI). *Red boxes* mark residues totally conserved in the three sequences and red residues boxed in *blue* are highly similar residues. **b** Ribbon representation of the HIV-1 protease dimer (2HS1) used as a main template in the modeling. Strands are *colored* in *yellow*, loop in *gray*, turn in *blue*, and α-helices in *red*. Active site residues are represented with their carbon atoms colored in *cyan*, nitrogen in *blue* and oxygen in *red*. **c** Model of HERV-K protease, carbon atoms of the conserved residues of the active site are colored *green* and non-conserved *red*

**Fig. 6:**
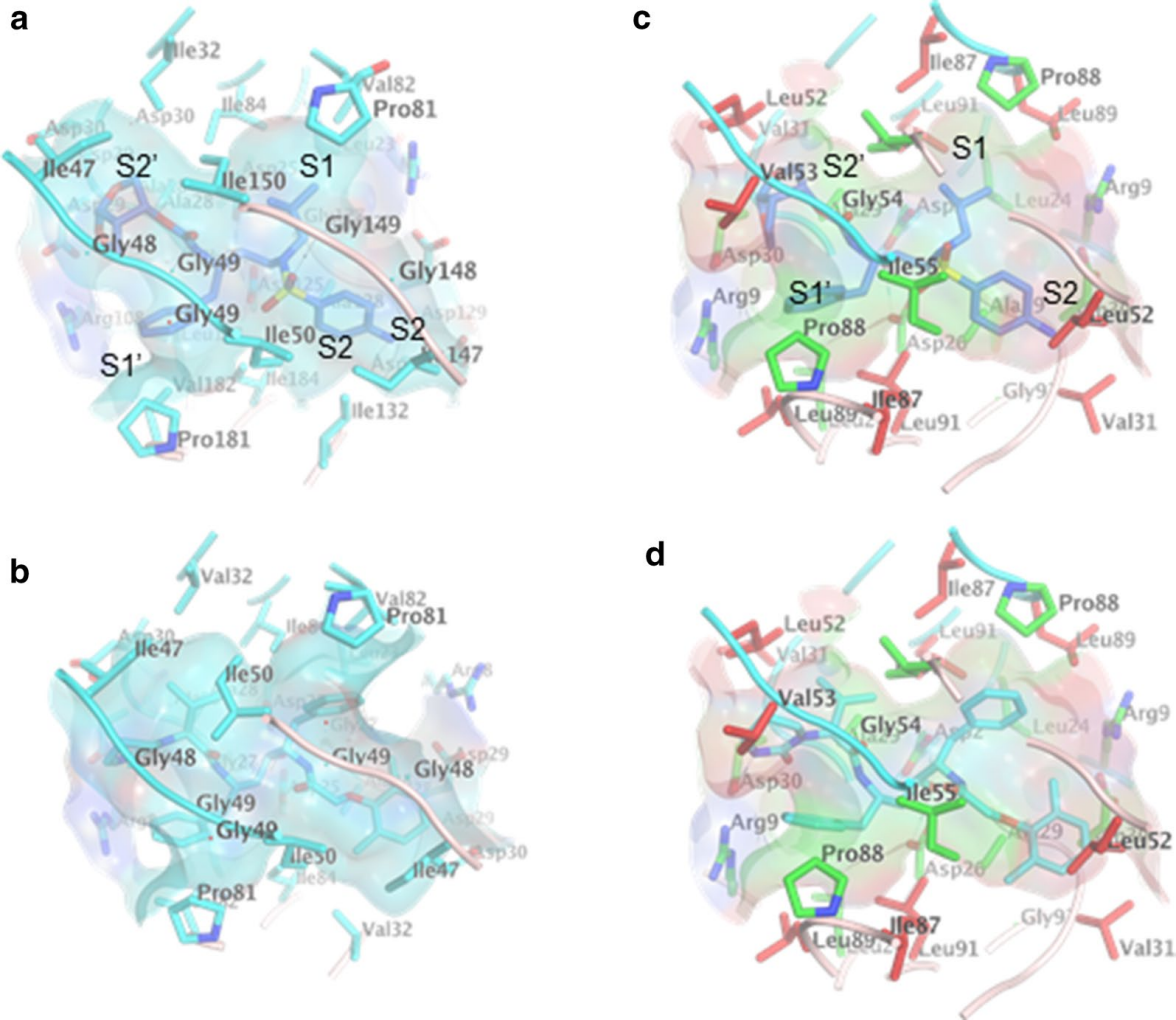
Protease inhibitors duranavir and lopinavir docked to the active site cavity of the viral proteases. **a** Darunavir (in *blue*) as in observed in the ultrahigh-resolution crystal structure (2HS1) and **b** Lopinavir (*cyan*) as bound to HIV-1 protease dimer. The semi-transparent surface of the active site cavity is shown using the color of the near atom. Ribbons are colored *cyan* and *pink* to represent the different monomers. **c**, **d** Models of these inhibitors docked to HERV-K protease. Carbon atoms of the HIV-1 protease are colored *cyan*, the conserved residues in HERV-K instead are colored *green* and the non-conserved *red*. Oxygen atoms are colored *red* and nitrogen *blue*

Although HERV-K protease has only 20% amino acid homology with HIV-1 protease (Fig. 5a), their core functional domains share similar structures. Comparative modeling of this core showed that the residues centered at the active site cavity and participating in the dimer interface are fully conserved between these proteases (Fig. 5b, c). The residues participating in the dimer contacts at the tip of the hairpin that forms the protease flap domain show high conservation. However HERV-K has an insertion in the N-terminal region of the flap that may impact its flexibility (Fig. 5c). This flexibility has been long recognized to play a role in inhibitor/substrate binding. At S1 (amino acids 87–91) and S2 (amino acids 30, 31, 52, 91) and their symmetry related S1′ and S2′ pockets, residue changes for larger residues reduce the size of the catalytic site. Most of these changes conserve hydrophobic characteristics at the site, only residue 31 changes from Asp to Val. At the S1 pocket, Leu89 and 91 replace HIV-1 smaller Val and Ile, respectively, reducing the size of this hydrophobic pocket (Fig. 5a, c). Val31, Leu53, Val53, and Leu91 replace Asp30, Ile47, Gly48, and Ile89 respectively reducing also the size of S2. However, the protease inhibitors Darunavir (Fig. 6a, c) and Lopinavir (Fig. 6b, d) readily dock to the protease catalytic site. This modeling suggests that HIV-1 proteases inhibitors should have also an inhibitory effect on HERV-K protease, although tweaking the size of the groups at these pockets could positively impact drug inhibition.

**Fig. 7:**
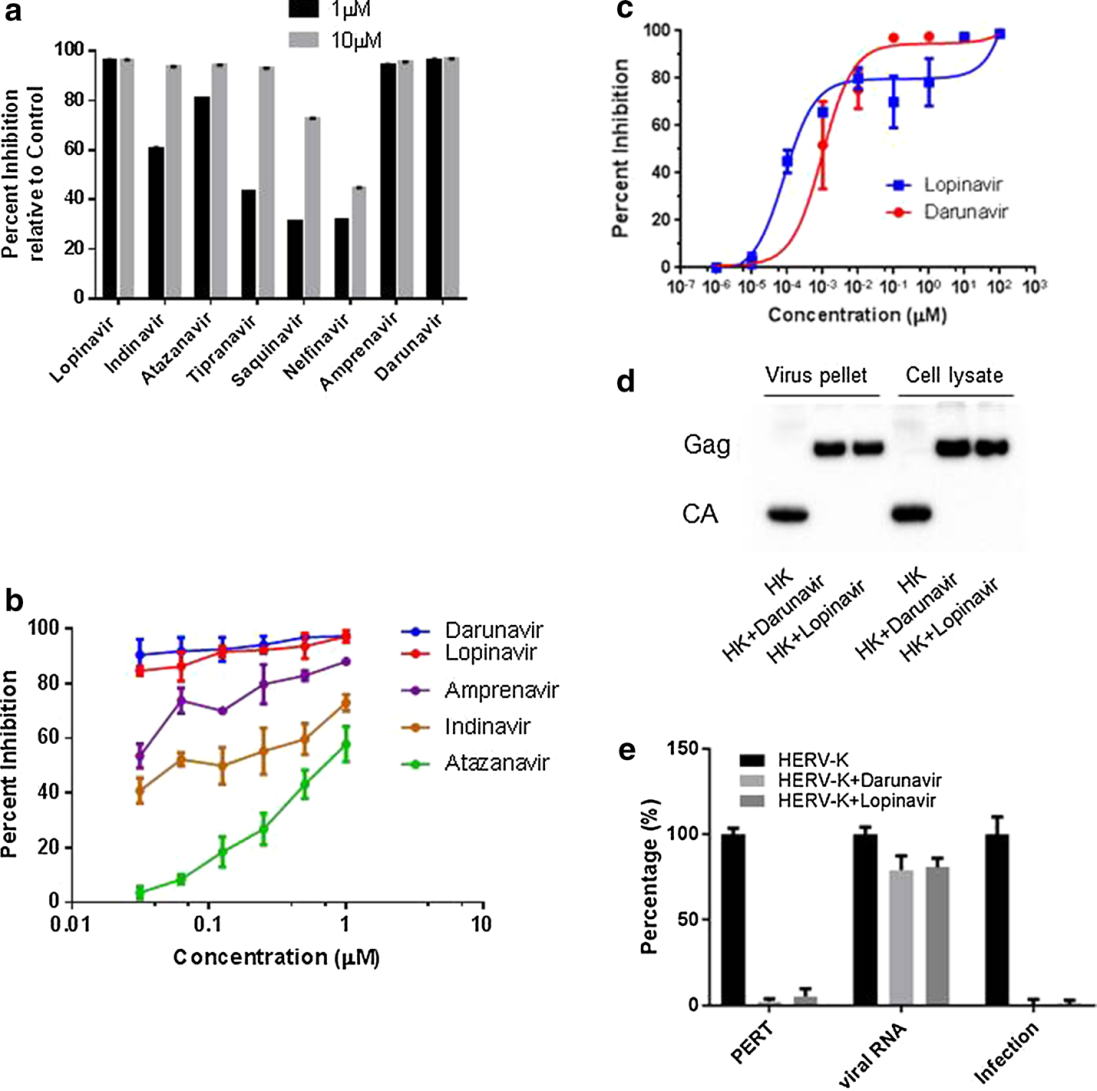
Protease inhibitors block replication of HERV-K viral particles: HeLa cells were transfected with pCD-HK/Rev and HIV-1 Tat plasmids. **a** HIV protease inhibitors were added to Hela cells 6 h post transfection and the reverse transcriptase (RT) activity in the culture supernatant was determined by PERT assay at 24 h post-treatment. **b** Darunavir, Lopinavir, Indinavir, Amprenavir or Atazanavir were added to HeLa cells 6 h post transfection in a twofold serial dilution ranging from 31.25 nM to 1 µM and RT activity in the culture supernatant was determined by PERT assay at 48 h post-treatment. **c** Darunavir and Lopinavir were further tested using tenfold serial dilution of the compounds, ranging from 100 nM to 100 µM. Viral supernatant was collected 48 h post-treatment and analyzed by PERT assay. Any change in Ct (threshold cycle) was compared to vehicle control and reported as percent inhibition. Data represent mean ± SEM of at least three different experiments. **d**, **e** 293 cells were transfected with pCD-HK/Rev in the presence or absence of 1 µM Darunavir or Lopinavir. After 48 h, HERV-K viral particles were harvested from the culture medium. The expression of Gag in the cell lysate and viral particles was determined by Western blot analysis. Darunavir and Lopinavir effectively blocked the processing of Gag (70 kDa) into mature capsid (CA) protein (27 kDa) both in the cell lysate and in the viral particles (**d**). Viral supernatant was analyzed by both PERT assay and viral RNA PCR, indicator of numbers of viral particles. The viral supernatant was also used to infect HeLa cells. After 6 days post-infection, HERV-K Gag mRNA in the infected cells was measured by PCR. Values from HERV-K with PI treatment were expressed as percentage of that without PI treatment

To determine the effect of protease inhibitors on HERV-K, HeLa cells were transfected with pCD-HK/Rev and HIV-1 Tat plasmids. HIV-1 protease inhibitors were added to the culture medium 6 h. after the transfection. PERT assay was performed 48 h after transfection. As shown in Fig. 7b, all protease inhibitors significantly inhibited functional HERV-K viral production in a dose-dependent manner. Lopinavir and Darunavir showed the highest efficacy, with IC_90_ in the 0.1 µM range. To further determine the efficacy of Lopinavir and Darunavir at lower dosages, more extensive dose–response curves were conducted. Darunavir and Lopinavir (Fig. 7c) both inhibited HERV-K protease in a dose-dependent manner, with IC_90_ of 0.071 and 0.651 µM respectively. To further determine the mechanism of HERV-K inhibition by protease inhibitors, 293 cells were transfected with pCD-HK/Rev in the presence or absence of protease inhibitors, Darunavir or Lopinavir at a concentration of 1 µM each. After 48 h, HERV-K viral particles were harvested from the culture medium. The expression of Gag protein in the cell lysate and viral particles was examined by Western blot analysis. Darunavir and Lopinavir effectively blocked the processing of Gag (70 kDa) into mature capsid (CA) protein (27 kDa) both in the cell lysate and in the viral particles (Fig. 7d). PERT assay and viral RNA PCR were performed to determine if functional RT activity correlates with the number of viral particles in the culture supernatant, As shown in Fig. 7e, PI treatment dramatically blocked RT activity as shown by the PERT assay, while the number of viral particles as inferred by PCR for viral RNA had only a moderate reduction. The slight reduction in the number of viral particles could be explained by the potential toxic effect of PIs on the cells and/or less efficient viral release due to lack of cleavage of the Gag precursor protein. To examine the effect of PI on HERV-K infection, the harvested viral particles with VSV-G pseudotyping were used to infect HeLa cells. After 6 days of infection, gag RNA expression was measured by PCR. HERV-K virus with PI treatment was unable to replicate compared to virus without PI treatment (Fig. 7e).

### Inhibition of HERV-K infection by integrase inhibitor

Currently there are three FDA-approved IN inhibitors: Dolutegravir, Elvitegravir, and Raltegravir. We tested the effect of Raltegravir on HERV-K replication. VSV-G pseudotyped HERV-K was used to infect HeLa cells. Raltegravir was added immediately after viral inoculation. After 6 days of infection, HERV-K *gag* gene was determined by qPCR. As shown in Fig. 3d, Raltegravir inhibited the replication of HERV-K in a dose-dependent manner, with an IC_90_ of 0.075 µM.

**Fig. 8:**
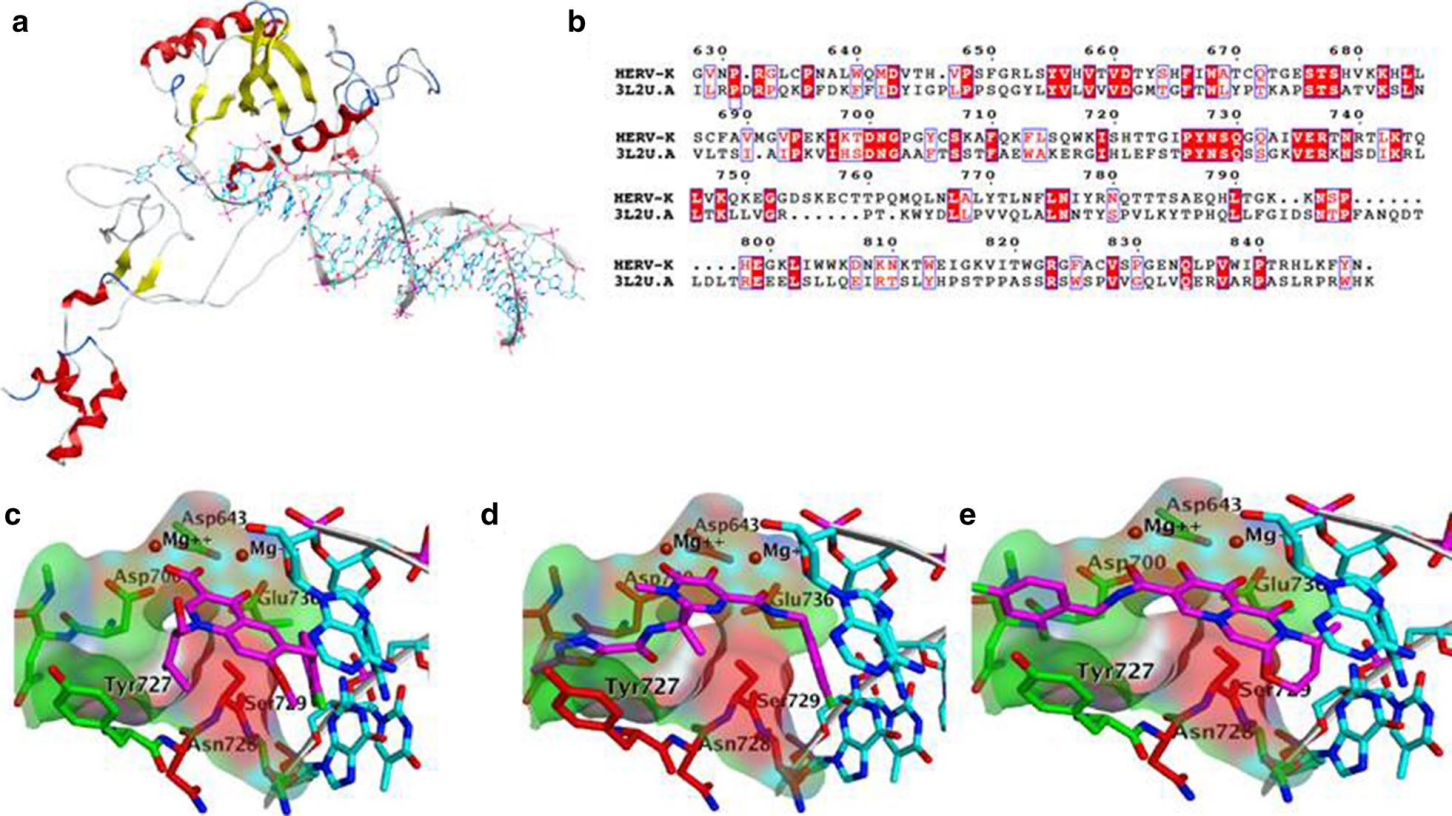
Comparative modeling of HERV-integrase active site. **a** Model of the integrase with the DNA bound. **b** Alignment between target and template was used in the modeling; *red boxed* residues are identical. **c**–**e** The integrase inhibitors mentioned bound to HERV-K integrase active site: **a** Elvitegravir, **b** Raltegravir were based on the crystal structure of their complexes of PFV (3L2U, 3L2V), and **c** Dolutegravir. Carbon atoms of the enzyme residues are colored *green* when they are conserved between PFV and HERV-K integrase and *red* when are not. DNA bases have their carbon atoms colored *cyan*

Comparative modeling of the HERV-K IN active site using simian prototype foamy virus (PFV) IN core domain as template (18% identity) [12] showed the conservation of the three carboxylated coordination of a pair of divalent metal cations (Mg^2+^ or Mn^2+^) that assist the nucleophilic substitution of the viral DNA (Fig. 8). Elvitegravir (3L3U) and raltegravir (3L2V) inhibit PFV INT [12]. The only active site difference between these enzymes, a proline to serine (Ser729) residue participates in these drugs recognition; however, the small change produced by the substitution could have little effect on the inhibitor recognition (Fig. 8).

## Discussion

We used a consensus sequence of HERV-K for these studies since there are nearly 100 copies of the virus in the human chromosome [8] and multiple loci of HERV-K are expressed in patients with ALS [2, 11]. Similarly in patients with HIV infection there is wide variability in viral sequences among individuals but drug development has been successful using laboratory strains of the virus.

Currently there are 26 antiretroviral drugs approved by the FDA. Suppression of HIV infection requires the use of combination therapy with RT inhibitors, protease inhibitors, and IN inhibitors.

The RT is critical for the replication of the viral RNA genome. In a reconstructed consensus HERV-K sequence, RT activity is critical for its replication [9, 13]. Inhibition with HIV RT inhibitors can significantly decrease the titer of infectious viral particles. RT has two enzymatic functions: DNA polymerase activity that synthesizes DNA from either a RNA or a DNA template, and RNase H activity that degrades the RNA strand in a RNA/DNA duplex. Because RT plays a central role in the viral life cycle, it is a key target for the development of antiretroviral drugs. There are two broad classes of RT inhibitors: nucleoside RT inhibitors (NRTIs) and non-nucleoside RT inhibitors (NNRTIs). NRTIs are structural analogs of the natural substrates of DNA polymerization. They compete with the natural substrates for incorporation in viral DNA by RT and terminate DNA chain extension because of the lack of the 3′-OH group. In this study, we tested the following NRTIs, tenofovir, abacavir, stavudine, lamivudine, and zidovudine for their ability to inhibit HERV-K RT activity and HERV-K replication. Although these drugs had very similar dose response curves, Abacavir seemed to be more potent against HERV-K compared to its effect on HIV (Table 1). In comparison, Zidovudine was slightly less potent against HERV-K compared to its effects of HIV [14]. This may be important since Abacavir and Zidovudine have better CNS penetration compared to the other NRTIs [15]. However Abacavir can cause a hypersensitivity syndrome which is strongly but not exclusively associated with a specific allele at the human leukocyte antigen B locus namely HLA-B*57:01 [16]. NNRTIs do not compete with the nucleoside substrate of RT, instead they bind to a hydrophobic pocket close to the polymerase active site called NNRTI-binding pocket [17, 18]. Binding of NNRTI to RT affects the alignment of the DNA chain terminus for extension with the RT catalytic site and interferes the chemical synthesis of viral DNA [19, 20]. Efavirenz, Etravirine, and Nevirapine are FDA-approved NNRTIs tested in this study. These compounds had similar efficacy against HERV-K however, of these compounds, Nevirapine has the best CNS penetration [15].

**Table 1:** Comparison of HIV and HERV-K IC90 values for antiretroviral drugs

Antiretroviral drugs	HIV IC_90_	HERV-K IC_90_ (µM)	HERV-K IC_50_ (µM)
Abacavir	2.3 µM	0.175	0.006
Zidovudine	0.03 µM	0.070	0.008
Darunavir	2.7–13 nM	0.071	0.029
Lopinavir	IC_50_ 17 nM (no serum) IC_50_ 102 nM (50% serum)	0.651	0.034
Raltegravir	0.033 µM	0.075	0.01

HIV protease is crucial for the maturation of viral particles. It is a homodimer and belongs to the aspartate protease family [21]. It cleaves Gag and Gag–Pol polyprotein precursor to produce capsid (CA) and active RT proteins. There are about 10 FDA-approved protease inhibitors. These inhibitors share similar chemical structures and similar binding property. They block the enzyme catalytic site by mimicking the transition state of the real substrate [21].

HERV-K protease is encoded in *pol* gene. It belongs to the aspartate protease family and includes a signature motif of aspartate–threonine–aspartate similar to HIV protease. Its core functional domain has about 106 amino acid residues and shares only 28% homology with HIV protease [22]. However modeling of the HERV-K protease shows similarities between the active domains with the HIV-protease. The protease inhibitors could be docked to the active domain of HERV-K protease. Consistent with these observations, we found that Darunavir and Lopinavir were able to inhibit HERV-K replication in a dose-responsive manner. However the IC_90_ of protease inhibitors (Darunavir and Lopinavir) for HERV-K were 20–50 times higher than that for HIV (Table 1). This supports the notion that protease inhibitors are more virus specific than RT inhibitors. A report using HERV-K10 protease also showed that HIV protease inhibitors were not as effective against HERV-K protease [22]. This suggests that HERV-K protease specific inhibitors may need to be discovered for effective antiretroviral therapy for HERV-K.

We found that IN inhibitor Raltegravir is highly effective against HERV-K, indicating that replication of HERV-K is integration dependent. IN is a key enzyme in the life cycle of the retrovirus and is coded in the *pol* gene. The catalytic core domain of HIV IN transfers the viral DNA into the chromosome with the help of both C- and N-terminal domains [23] HIV IN has similar structural similarity with other retroviral INs from avian sarcoma virus, rous sarcoma virus and simian immunodeficiency virus, indicating they may share a similar mechanism of action [23]. Comparative modeling showed that there are almost no differences in the active site of HERV-K and simian PFV INs that can be inhibited by Raltegravir [12]. The inhibitors target the DNA stand transfer process. Hence they are called strand transfer inhibitors. Raltegravir is one of the FDA approved stand transfer inhibitors and is highly potent in inhibiting both HIV and HERV-K replication (Table 1). Because IN inhibitors have less side effects compared to RT and protease inhibitors, it should be an important component in any antiretroviral regime.

Our data suggests that similar to HIV antiretroviral treatment, complete inhibition of HERV-K may require combination therapy that targets different parts of the life cycle of the virus particularly since most of these drugs are less potent in their activity against HERV-K compared to their effects on HIV. However, studies are needed to determine the efficacy of combined inhibitors on HERV-K replication. Currently, it is not clear if the activation of HERV-K expression in ALS patients leads to production of infectious viral particles. The anitretrovirals will be effective only if there was active infection and replication. For treatment of ALS patients with antiretroviral drugs, the drugs need to have good blood brain barrier penetration to target brain neurons with increased HERV-K expression.

## Conclusions

We identified that FDA approved RT and IN inhibitors can effectively inhibit HERV-K virus, while protease inhibitors were not as effective in inhibiting HERV-K virus as HIV. Development of new protease inhibitors for HERV-K may be required. These antiretrovirals may open new prospects for studying HERV-K pathophysiology and potentially for exploring treatment of diseases in which HERV-K has been implicated.

## Methods

### DNA constructs and HIV inhibitors

HERV-K whole genome consensus sequence [1] was synthesized and cloned into pcDNA3.1 vector (Invitrogen). HIV-1 Rev plasmid was reported previously [2]. To increase the production of HERV-K viral particles, the Rev expression cassette was inserted to the pcDNA3.1-HERV-K construct. The resulting plasmid was called pCD-HK/Rev. All HIV inhibitors and VSV-G plasmid were obtained from NIH AIDS reagent program (http://www.aidsreagent.org). A stock of 10 mM was made by diluting the inhibitors in dimethyl sulfoxide (DMSO). For further use serial dilutions for each inhibitor was made in complete media: Dulbecco’s modified Eagle’s medium; DMEM + 10% fetal bovine serum (FBS) and penicillin–streptomycin.

### Cell culture and transfection

The human cell lines 293T and Hela were maintained in DMEM supplemented with 10% FBS and penicillin–streptomycin. For testing activity of HIV-RT inhibitors in a cell free system, Hela cells were transiently transfected with pCD-HK/Rev in 24-well plates at 0.2 × 10^6^ cells/well using lipofectamine 2000 (Invitrogen) according to the manufacture’s protocol. Virus particle-containing supernatants were collected after 24 and 48 h. Control experiments included mock transfection with empty vector pcDNA3.1. Cell culture supernatants were assayed for RT activity using a PERT assay as described below. At the time of reverse transcription, HIV nucleoside or non-nucleotide RT Inhibitors were added to the supernatant at six different doses ranging from 0.001 to 0.25 µM. Any change in RT activity was expressed as percent inhibition relative to no treatment control.

For testing the activity of HIV protease inhibitors against HERV-K, HeLa cells were transiently transfected with pCD-HK/Rev as described above. Six hours post-transfection, culture medium was completely replaced with fresh medium containing HIV protease inhibitors in a twofold serial dilution ranging from 31.25 nM to 1 µM. After 48 h, cell culture supernatants were collected and RT activity in the culture supernatant was determined by PERT assay. Darunavir and Lopinavir were identified as the two most potent drugs and were further screened in a tenfold-serial dilution treatment ranging from 0.001 to 100 µM.

### Recombinant virus production and infection

293T cells were cultured in DMEM with 10% FBS and penicillin–streptomycin. Cells were transiently transfected in 10 cm plates at 5 × 10^6^ cells/plate using lipofectamine 2000 (Invitrogen) according to the manufacturer’s protocol. Briefly, cells were co-transfected with pCD-HK/Rev with or without pCD-VSV-G. After 24 h, the transfection medium was completely removed and cells were washed with phosphate-buffered saline (PBS) to eliminate any residual plasmid and then fresh medium was added to the cells. Virus particle-containing supernatants were harvested after an additional 24–48 h and cleared of any cellular debris with two centrifugations at 1000×*g* at 4 °C. The clarified samples were then subjected to DNase treatment using the RNase-free DNase kit (Qiagen). Cleared supernatant was concentrated using Retro-X™ Concentrator (Clontech) as per manufacturer’s instructions. Briefly, viral supernatant was mixed with the Retro-X Concentrator and incubated overnight at 4 °C. The mixture was then centrifuged at 1500×*g* for 45 min at 4 °C to obtain a virus-containing pellet. The viral pellet was gently resuspended using complete DMEM and titrated using the PERT assay. An absolute amount of RT was determined using HIV RT as standard and 80 pg of HERV-K virus was used for each infection. At the time of transduction of target cells, the concentrated virus was again treated with RNase free DNase to ensure there was no plasmid DNA contamination. Infection was performed by exposing the resuspended DNase treated viral samples with fresh 293T or HeLa cells that had been plated in 24-well plates 24 h earlier in 5 μg/ml of polybrene in the presence or absence of Abacavir, Zidovudine or Raltegravir. Total RNA was extracted 6 days post infection and HERV-K Gag gene expression was quantified using QPCR. Any change in RT inhibitor treated wells compared to untreated was expressed as percent inhibition. To determine the effect of protease inhibitors on HERV-K infection, 293T cells were transfected with pCD-HK/Rev and pCD-VSV-G in the presence of 1 µM of Darunavir or Lopinavir. After 24 h, the transfection medium was completely removed and cells were washed with PBS. Fresh medium with 1 µM of Darunavir or Lopinavir was then added back. After another 24 h, viral particles were harvested and used to infect 293T cells using the same method mentioned above, but without any further PI treatment during infection.

### RNA extraction and quantitative PCR

Total RNA was extracted from cultured cells with RNeasy Plus mini kit (Qiagen) and treated with RNase-free DNase. Reverse transcription was performed with 500 ng RNA using Superscript III first stand synthesis Supermix kit (Invitrogen). Gene expression levels for HERV-K gag and glyceraldehyde 3-phosphotate dehydrogenase (GAPDH) were determined by quantitative PCR performed on an ABI PRISM 7000 Sequence Detection System (Applied Biosystem, Carlsbad, California). PCR primers are listed in Table 2. Absence of DNA contamination in the RNA preparation was confirmed when target gene was not detected by PCR when RT was omitted during reverse transcription. The amount of RNA was expressed as fold change using GAPDH as an internal standard.

**Table 2:** PCR primers

Target gene	Primer sequence (5′–3′)
HERV-K env	Forward: CTGAGGCAATTGCAGGAGTT
	Reverse: GCTGTCTCTTCGGAGCTGTT
HERV-K gag	Forward: AGCAGGTCAGGTGCCTGTAACATT
	Reverse: TGGTGCCGTAGGATTAAGTCTCCT
GAPDH	Forward: TGCACCACCAACTGCTTAGC
	Reverse: GGCATGGACTGTGGTCATGAG
MS2	Forward: TCCTGCTCAACTTCCTGTCGA
	Reverse: CACAGGTCAAACCTCCTAGGAATG
	Probe: [6FAM]CGAGACGCTACCATGGCTATCGCTGTAG[TAM]

### Product enhanced reverse transcriptase (PERT) assay

PERT assay was used as described [3] with minor modifications. Briefly, cell culture supernatant was collected and centrifuged to pellet any cell debris. The cleared supernatant was then supplemented with 0.25% Triton X-100, 5 mM dithiothreitol and 0.25 mM ethylene-diamine-tetra-acetate as the source of HERV-K RT. Bacteriophage MS2 genomic RNA was used as template for the reverse transcription reaction. Quantitative PCR was performed with TaqMan primers (MS2-Forward and MS2-Reverse) and probe (MS2-Probe) using Applied Biosystems Vii 7. RT activity was expressed as fold change compared to control or as pg/ml RT determined by standard curve generated from PERT using HIV-1 RT.

### Western blot analysis, immunoflourescence and antibody production

For Western blot analysis of HERV-K viral protein expression and cleavage, 293T cells were transiently transfected with either the HERV-K expression vectors or empty vector using lipofectamine 2000 (Invitrogen). After 48 h transfection, cells were washed with PBS and lysed in radioimmunoprecipitation assay (RIPA) buffer containing protease inhibitors (Roche). The insoluble pellet was removed by a 10 min centrifugation at 12,000×*g*. The harvested lysates were separated by sodium dodecyl sulfate polyacrylamide gel electrophoresis using Novex 4–12% Bis–Tris gels (Invitrogen), followed by transfer onto polyvinylidene fluoride membranes. The blots were incubated overnight at 4 °C with either anti-HERV K Env antibody (Austral biologicals) or anti HERV K Gag antibody (Austral biologicals) followed by 1 h incubation with a secondary antibody linked to horseradish peroxidase. After 30 min washing, the blot was developed with SuperSignal™ West Femto ECL reagent (ThermoFisher), and imaged with FluoroM imaging machine (ProteinSimple). Immunofluorescence analysis for the co-localization of Gag and Pol was performed on 293T cells transiently transfected with the HERV-K expression vector or empty vector (negative control). Twenty-four hours post-transfection cells were fixed with 4% paraformaldehyde, permeabilized, and stained with a rabbit polyclonal anti-Pol serum and a mouse monoclonal anti-Gag antibody. Alexa 488-conjugated goat anti-rabbit IgG and Alexa 594-conjugated goat anti-mouse IgG were used as secondary antibodies (molecular probes); nuclei were stained with 4′,6-diamidino-2-phenylindole (DAPI; molecular probes).

A polyclonal anti-HERV-K Pol antibody using amino acids 57–245 as an immunogen was developed by SDIX with its proprietary Advanced GAT technologies. The monoclonal antibodies against the full length HERV-K Env and Gag were obtained from Austral Biologicals. Rabbit antisera against HERV-K envelope protein were developed by Genscript, using peptides QRKAPPRRRRHRNRC, CSDLTESLDKHKHKK, and CSKRKGGNVGKSKRD as immunogens.

### Toxicity assay

HeLa cells were cultured in microplates (tissue culture grade, 96 wells, flat bottom) in a final volume of 100 µl/well culture medium in a humidified atmosphere (e.g., 37 °C, 5% CO_2_). 24 h later, the cells were treated with HIV inhibitors at dosage ranging from 0.01 to 10 µM. Six days post-treatment Cell Proliferation Reagent WST-1(Roche) was used per manufacturer’s instructions to determine drug toxicity. Briefly, 10 µl of Cell Proliferation Reagent WST-1 was added to each well and the plate was shaken thoroughly for 1 min on a shaker. The cells were incubated for 0.5–4 h in a humidified atmosphere (37 °C, 5% CO_2_). The absorbance of the samples against a background control as blank was measured using a microplate reader at 420–480 nm using a FlexStation microplate reader (molecular devices).

### Comparative modeling

All the comparative modeling was performed using the homology modeling protocols implemented in the program molecular operating environment (MOE) [22]. The sequences of target and templates were initially aligned with clustalW [23] and manually adjusted after inspection to place insertions and deletions in favorable regions. An AMBER10HT force field was used for energy calculations and minimization. Ramachandran’ plot showed 95% of the residues of the final model are in allowed regions, and no rotamer outliers are present. When complexes between template and target inhibitors are available, the poses of the inhibitors in HERV-K targets were based on that of the template’s complexes. When required to improve the pose, rotamers of selected residues 4.5 Å apart from the drug were explored to relieve the few clashes observed and to improve contacts, as well as drug and nearby residues relaxed by minimization. The drug minimization in the active site environment of the HERV-K targets was performed, tethering the protein atoms to their initial position with a weak harmonic potential (0.5 kCal/mol) during minimization.

In the case of the protease, the structures of a dimer of HIV-1 (PDBId: 2HS1, 0.85 Å) and Rous sarcoma virus (RSV; 1BAI, 2.4 Å) proteases were used as templates. The RSV protease structure was used to model the insertion in the loop between β4–5 because it displays similar characteristics to the one in HERV-K protease and, the HIV-1 protease was used for the rest of the model (Fig. 4b). Three features common to retroviral proteases were carefully maintained in the alignment and model: (1) the active site triad (26-DTG-28), (2) the highly conserved triad GRN/D unique to retroviral proteases [24], and (3) the intra- and inter-subunit salt bridge between R89, D30, and R9′. To model complexes of HERV-K protease with inhibitors the structures of the highest resolution complexes of the HIV-1 protease with Lopinavir (2OS4), and Darunavir (2HS1) were used. Models of the complexes with Darunavir and Lopinavir were prepared by overlaying the respective complexes structures with the HERV-K model.

In the case of the HERV-K RT, HIV-1 RT crystal structure (4W1E) was used as template, and the crystal structures of the complexes with Efavirenz (1JKH), Nevirapine (3QIP), and Etravirine (3MEC) were used to model the inhibitors bound to HERV-K RT.

In the case of the HERV-K IN the simian prototypical foamy virus in complex with magnesium, DNA, and Elvitegravir (3L2U) with an 18% of identity with the target was used as template. This elvitegravir complex and the complexes with ratelgravir (3L2) and dolutegravir (3S3M) were used to model these inhibitors complexes with HERV-K IN (Fig. 8c–e).

### Data analysis and statistics

All the experiments were repeated at least three times. Representative results were shown and plotted as mean ± SEM. Student’s t test was used for pair-wise comparison.
